# Can father inclusive practice reduce paternal postnatal anxiety? A repeated measures cohort study using the hospital anxiety and depression scale

**DOI:** 10.1186/1471-2393-12-75

**Published:** 2012-07-31

**Authors:** Jenny Tohotoa, Bruce Maycock, Yvonne L Hauck, Satvinder Dhaliwal, Peter Howat, Sharyn Burns, Colin W Binns

**Affiliations:** 1 School of Public Health, Curtin Health Innovation Research Institute, Curtin University, Perth, WA, Australia; 2 School of Nursing and Midwifery, Curtin Health Innovation Research Institute, Curtin University, Perth, WA, Australia; 3 Centre for Behavioural Research Cancer Control, Shenton Park, WA, Australia

## Abstract

**Background:**

Perinatal research on anxiety and depression has primarily focused on mothers. We have limited knowledge of fathers’ anxiety during the perinatal period yet there is evidence that the parenting capacity of a person can be compromised by anxiety and depression. The purpose of this paper is to identify the impact of a father inclusive intervention on perinatal anxiety and depression. The prime focus of the intervention was to provide education and support to fathers of breastfeeding partners with the aim of increasing both initiation and duration of breastfeeding.

**Methods:**

A repeated measures cohort study was conducted during a RCT that was implemented across eight public maternity hospitals in Perth, Western Australia between May 2008 and June 2009. A baseline questionnaire which included the Hospital Anxiety and Depression Scale (HADS) was administered to all participants on the first night of their hospital based antenatal education program and was repeated at six weeks postnatal. SPSS version 17 was used for reporting descriptive results.

**Results:**

The mean anxiety levels at baseline for the fathers in the intervention group (n=289) and control group (n=244) were 4.58 and 4.22 respectively. At 6 weeks postnatal (only matched pairs), intervention and control group were 3.93 and 3.79. More intervention group fathers self-rated less anxiety compared to the fathers in the control group from baseline to post test (p=0.048). Depression scores for intervention fathers at baseline (mean =1.09) and at six weeks (mean=1.09) were very similar to fathers in the control group at baseline (mean=1.11) and at six weeks (mean =1.07) with no significant changes.

**Conclusions:**

Both intervention and control group fathers experienced some anxiety prior to the birth of their baby, but this was rapidly reduced at six weeks. Paternal anxiety is common to new fathers and providing them with information and strategies for problem-solving can increase their knowledge and potentially lower the risk of postnatal anxiety.

**Trial registration:**

(Australian New Zealand Clinical Trials Registry ACTRN12609000667213)

## Background

Much research has explored postnatal depression in women [1-5] with less research on anxiety [6-9]. However, there is limited evidence on paternal postnatal anxiety and depression [10]. The changing role of fathers over the past two decades in developed countries has seen a shift from men being primarily breadwinners to fathers being expected to actively participate in nurturing and caring for their children [11]. This shifting role attribution has been associated with reduced self confidence and increasing anxiety and depression in men already feeling overwhelmed with the transition to parenthood [12-14]. The joy and excitement of becoming a new father is often accompanied by an increase in anxiety and apprehension [15].

Fathers have an important contribution to make to the ongoing emotional, mental and physical development of their children [16][17]. The importance of reducing parental anxiety and the impact of antenatal distress, depression and anxiety on infant outcome levels have been documented in several studies [18][19,20]. A father’s anxiety (assessed with the adult ADIS [Anxiety Disorder Interview Schedule]) can influence the anxiety levels of his child [21]. The potential changes in lifestyle and interpersonal relationships, increased financial concerns and embracing a different self-identity can exacerbate anxiety and depression for some new fathers [22,23]. Condon and colleagues (2004) found the lack of understanding of what is expected of a father might cause anxiety, especially for first-time fathers and lead to a greater risk of paternal depression [22].

Men need information that is relevant to their new role of father [24] and hospital based antenatal programs are one place this information could be delivered. However, antenatal classes can vary in the quality of the content and delivery of information [23]. Most antenatal classes in Australia are coordinated between midwives and physiotherapists and focus on the birth process, pain control and the role of the child health nurse in the community [25].

Anxiety is defined as a psychological and physiological state characterized by cognitive, somatic, emotional, and behavioural components [26]. It can be generalised, episodic: pregnancy or exam time; or specific: phobias and obsessive compulsive disorder. Depression is defined in terms of a specific alteration in mood continued over two weeks or more with a negative self-concept associated with self-reproach and self-blame, anorexia, insomnia and a change in activity level [27].

In a general population depression and anxiety frequently present together in the same individual and diagnosing and treating these conditions is paramount to improved mental health and functioning [28]. Mild depression has been linked to co-morbid anxiety and over time can convert to major depression [29]. The relationship between parenting stress and postnatal depression appears to be a reciprocal one with each contributing to the other [3]. Several studies have found that first-time fathers reported higher levels of anxiety during the early postnatal period [30,31], and around 10% reporting a significant elevation of anxiety levels [32]. An American study researching the role of fathers in child anxiety, found that paternal attachment might be an important protective factor in decreasing the development of child anxiety, and that paternal anxiety could decrease attachment [33]. In an Australian study with 356 fathers, Matthey et al., (2003) found that men had a significantly increased chance of getting depressed if they had anxiety problems [10]. In the Avon Longitudinal Study of Parents and Children (ALSPAC) study conducted in Britain, 10,975 fathers and their children were followed up for 7 years. They found that children whose fathers were depressed in both the antenatal and postnatal periods presented with higher risk of early behavioural and emotional development problems [34].

Most studies of parental anxiety and depression use the Edinburgh Postpartum Depression Scale (EPDS), and focus on the mother and the mother-infant dyad with little attention to paternal anxiety and depression [1,35-37]. In Australia, most pregnant women attending a public health service are routinely screened for depression at their antenatal clinics or with their doctor using the EDPS [38]. Whilst men are not routinely screened for depression or anxiety in Australia, the EDPS has been used successfully with 157 couples by Matthey et al., (2000) to determine mood disturbances for both mothers and fathers in the perinatal period [39]. Another tool for screening depression and anxiety in the primary care setting or community setting is the Hospital Anxiety and Depression Scale (HADS). Both anxiety and depression are identified as independent measures [40]. The HADS has recognised validity [41] and reliability [42] and is easy to administer [39]. The correlations between the two subscales varied from .40 to .74 (mean .56). Cronbach's alpha for HADS-A (anxiety) varied from .68 to .93 (mean .83) and for HADS-D (depression) from .67 to .90 (mean .82) [43]. Robertson et al., (2004) in their critical appraisal of the literature on maternal and paternal depression revealed a number of methodological and knowledge gaps including the use of appropriate instruments assessing postnatal depression for use within different cultural groups [44]. The HADS was chosen for this project as it was found to perform well in assessing the symptom severity of anxiety disorders and depression in both somatic, psychiatric and primary care patients and in the general population [43,45]. It has the advantage of evaluating both depression and anxiety and has been effectively used as a preliminary screening instrument in an antenatal population [46,47].

Risk factors for postnatal depression include marital dissatisfaction, antenatal life events, past depression, late antenatal depressive symptoms and lack of social support [48,49]. Although several studies have explored postnatal depression in fathers few studies have focussed upon the stress and anxiety that many men experience in their transition to parenthood. Maternal antenatal anxiety and depression occur frequently and often together and may lead to postnatal depression and anxiety. Paternal depression shows a moderate positive correlation with maternal depression [9,50]. Ballard and Davies (1996) found that paternal depression was associated with maternal depression, an unsupportive relationship and unemployment [51]. These findings were supported in a recent Spanish cross sectional study of 669 couples that found men with low social support and a partner with depression had an increased chance of becoming depressed themselves [52]. Depression in fathers during the postnatal period was associated with adverse emotional and behavioural outcomes in children aged 3.5 years (adjusted odds ratio 2.09, 95% CI 1.42-3.08), and an increased risk of conduct problems in boys (2.66, 1.67-4.25) [53]. Parent–child relationship quality appears to be a robust predictor of children's psychological development [54] and paternal postnatal depression is also a risk factor for child maltreatment and infanticide [55]. Interventions to address these risk factors have primarily focussed on the mother, [49,56] although a study undertaken by Davey et al., (2006) found men who attended a treatment program for their partners with postnatal depression, also valued the opportunity to share experiences with peers, hear strategies for engaging in their relationship, and gain factual information about postnatal concerns and apprehensions [57].

In an Australian longitudinal repeated measures study, 225 fathers were assessed for distress using the EPDS in early pregnancy, immediately post birth and at 4 months postnatal. They found that although the majority of men’s anxiety decreased steadily some struggled with their new role and responsibilities [58]. There is a gap in knowledge regarding paternal anxiety and depression and strategies to reduce postnatal anxiety. The purpose of this paper was to discuss the impact of a gender specific antenatal education session with follow up socio-educational support on anxiety and depression levels in the intervention fathers compared with the control group of fathers. This paper compared the paternal anxiety and depression scores (measured by HADS) in and between the intervention and control group of fathers prior to the birth of their child and at six weeks postnatal. Whilst the primary purpose of the intervention was to enhance fathers support for their partners breastfeeding and hence the duration of breastfeeding, we theorised that as the intervention contained many of the elements identified in the anxiety and depression literature as important to male participants (role exploration, anticipation of changes, development of strategies and the opportunity to discuss with other men their experiences and concerns) it would also reduce paternal anxiety.

## Methods

### Study design

A RCT to increase the duration of breastfeeding was conducted in eight public maternity hospitals in metropolitan Western Australia from 2008–2009. The intervention utilised father inclusive practice consisting of an antenatal education session led by a male facilitator, followed by a six week postnatal social support/education intervention consisting of education and support materials that were sent to the fathers at predetermined times [59]. As part of that trial a repeated measures cohort study was conducted to identify changes in self reported levels of anxiety and depression between the men in the intervention group and the men in the control group from baseline to six weeks.

A total of 1574 (862 women: 712 men) participants were recruited between May 2008 and July 2009. For fathers to be eligible to participate they had to be contactable by telephone or email at home or in the community; reside within Western Australia; and intend to participate in the rearing of their child. Participants were randomised to either intervention or control groups within each hospital without blinding. The demographic details of fathers who participated in the study are presented in Table 1 and highlight the homogeneity of the two groups. There were differences in the number of completed responses from fathers to each demographic question and also in the response rate for the anxiety and depression scores from fathers in the intervention and control groups both at baseline and at six weeks. Missing values are included in all tables.

**Table 1:** Demographics of fathers participating in the RCT

**Variable**	**Control N (%)**	**Intervention N (%)**
AGE	29.4 (17–54)	29.5 (16–51)
MARITAL STATUS	N=316	N=365
Single	21 (7%)	30 (8%)
Married	155 (49%)	200 (55%)
Defacto	130 (41%)	117 (32%)
Missing Value	10 (3%)	18 (5%)
EDUCATION	N =321	N =371
≤ year 12	125 (39%)	146 (39%)
Trade/diploma	122 (38%)	140 (38%)
Degree/higher	67 (21%)	70 (19%)
Missing Value	6 (2%)	15 (4%)
INCOME	N =300	N=350
≤$30,000	12 (4%)	14 (4%)
$31,000-$60,000	60 (20%)	70 (20%)
$61,000-$90,000	84 (28%)	105 (30%)
≥ $91,000	120 (40%)	130 (37%)
Missing Value	24 (8%)	31 (9%)
AUSTRALIAN BORN	215 (66%)	247 (65%)
RETURN TO WORK	N =298	N=353
≥ 1 week	125 (42%)	116 (33%)
<1 weeks>4 weeks	95 (32%)	148 (42%)
≤ 4 weeks	50 (17%)	56 (16%)
Missing Value	28 (9%)	33 (9%)
EMPLOYMENT	N=324	N=371
Full time	275 (85%)	326 (88%)
Unemployed	26 (8%)	15 (4%)
Other	20 (6%)	15 (4%)
Missing Value	3 (1%)	15 (4%)

Formative research data collected from mothers and fathers determined the content of the antenatal session and postnatal education/support package. This involved focus groups and an online survey [24]. The antenatal education session was underpinned by the Social Cognitive Theory, the Health Belief Model and Gender Theory [59]. The use of adult learning principles were used to increase health literacy [60,61] and problem-solving strategies and resources were incorporated into the antenatal session and follow up support to increase self-efficacy [59].

### Procedure

All expectant parents were sent an information letter about the RCT when they registered for their antenatal classes. On the first night of antenatal classes, each participant was invited to complete a consent form prior to answering the baseline questionnaire, with the option of withdrawing without penalty at any time. A short overview of the all-male group process and the follow up education/social support package was provided to those couples in the intervention group. The control group participated in the routine antenatal classes held at each of the hospitals incorporating labour, birth, pain relief and breastfeeding. All participants were sent the six week questionnaire by post, email or were contacted by phone for their responses.

The intervention group of fathers attended the routine antenatal classes with an additional one hour session incorporated into the hospital program. These sessions were facilitated by a male educator and addressed three main topics: the role of the father, the importance and benefits of breastfeeding for both mother and baby and what to expect in the first four weeks at home with a new baby. The details of the intervention have been reported elsewhere [59]. This was followed by a six week social and educational support package commencing from the birth of their baby and aimed to enhance the support for their partner’s breastfeeding. Information about developmental milestones, stress reduction strategies and postnatal depression were just three of the educational resources sent to the fathers.

### Data collection and analysis

Participants involved in the study completed a baseline questionnaire that included demographic data of age, marital status, nationality, income and educational level, plus the Hospital Anxiety and Depression Scale. At six weeks a short questionnaire was sent to all participants that identified the method of birth and any complications, current feeding practice and repeated the Hospital Anxiety and Depression Scale. For those fathers in the intervention, a series of evaluation questions on the six week support package were also asked.

The HADS is a self-report 14 question survey [43] used to identify anxiety and depression. The questionnaire has seven questions reflecting anxiety alternating with seven reflecting depression: HADS-A and HADS-D. Each item is answered on a four point (0–3) response category so the possible scores ranged from 0 to 21 for anxiety and 0 to 21 for depression. Several questions were reversed scored. A score of 0 to 7 for either subscale could be regarded as being in the normal range, a score of 8 to 10 being suggestive of either a mild anxiety or depression , a score of 11–14 a moderate degree of anxiety or depression and 15 and above indicating a more severe anxiety or depression. If participants registered scores of anxiety or depression above 14 the clinical nurse specialist at the relevant hospital was contacted and asked to follow up. All participants were aware of this safety net.

The anxiety levels (normal, mild, moderate and severe) data at baseline recorded by the intervention and control groups was compared to the levels recorded at 6 weeks (only matched pairs) using McNemar-Bowker’s test. The SPSS version 17 statistical package was used to analyse the data. Pearson’s Chi-square test was used with asymmetric 2 tailed tests for within group results and Fischer’s exact test was used for between group results.

Ethics approval for the RCT was granted from Curtin University and each of the hospital Health Services involved and informed consent was obtained from each participant prior to them completing any questionnaire. The Helsinki Declaration was upheld.

## Results

A total of 712 expectant fathers were recruited and 680 (95%): 315 (control), 365 (intervention) completed the HADS at baseline. At six weeks 556 (78%) fathers completed the HADS: 253 (control) and 303 (intervention). The change towards lower anxiety levels from baseline to six weeks were significant (p = 0.012) in the intervention group but were not significant in the control group (p = 0.410). The number of intervention fathers at baseline was 24 (7%) whilst 13 (4%) control fathers registered moderate to severe anxiety. At six weeks the anxiety levels fell for both groups of fathers with only 8 (2.6%) intervention fathers and 6 (2.4%) control fathers recording moderate anxiety. The attrition rate of 25% from the six week responses of both intervention and control fathers incorporated those participants who did not answer all seven anxiety and depression questions and those who did not complete the questionnaire. All participants were contacted up to three times over six weeks (in an effort to increase response rate) if they did not return the initial six week survey within three weeks.

The results are tabled to show the percentage of those fathers who improved their scores (lowered their self-reported anxiety) from baseline to six weeks. The number of fathers whose anxiety increased at six weeks was also lower in the intervention group and these results are recorded in Table 2. Missing values are included and represent those fathers who declined to respond or who did not complete the questions related to the anxiety and depression scale.

**Table 2:** Comparison of perinatal paternal anxiety scores for fathers in the intervention and control group of the RCT

**ANXIETY**	**Unchanged**	**Reduced**	**Increased**	**Total**
	**N (%)**	**N (%)**	**N (%)**	
Control (p=0.471)	198(81%)	28(11.4%)	18(7%)	244
Intervention (p=0.012)	241(83%)	36(12.4%**)	12(4%)	289
Total	475(82%)	64(11.9%)	30(5.7%)	533
Missing values				147

** p is significant at 0.015 using McNemar-Bowker test.

In the intervention group 12.4% of the fathers had less anxiety (self-reported) from baseline to six weeks postnatal compared to 11.4% of fathers in the control group. Comparison between the two groups found a marginal statistical difference to a lower anxiety level at six weeks postnatal (p = 0.048) using Fischer’s exact test.

Although both the intervention and control groups reported lower antenatal anxiety at six weeks postpartum the antenatal and postnatal depression scores remained unchanged at 4% in both groups see Table 3. Depression scores for the intervention fathers at baseline (mean =1.09) and at six weeks (mean = 1.09) were very similar to the fathers in the control group at baseline (1.11) and at six weeks (1.07).

**Table 3:** Comparison of perinatal paternal depression scores for fathers in the intervention and control group of the RCT

**DEPRESSION**	**Unchanged**	**Reduced**	**Increased**	**Total**
	**N (%)**	**N (%)**	**N (%)**	
Control (p=0.632)	227(91%)	11(4.4%)	11(4.4%)	249
Intervention (p=0.789)	263(90.3%)	14(4.8%)	14(4.8%)	291
Total	490(90.7%)	25(4.6%)	25(4.6%)	540
Missing values				140

Process evaluation of the father’s antenatal education session indicated a positive response to both the content and the facilitators. Positive feedback (96%) from fathers in the intervention group included comments like: “give[s] you a little insight to what you can expect coming out of hospital [and] how much your role changes”; “practical information on what to do as most of us haven't got any idea what’s going on”; “very useful presentation even for 2nd time dad “. Increasing awareness and knowledge was deemed important, as these participants stated: “every piece of advice and information you can get helps you prepare and make decisions later on”;“helped me with answers”; “really enjoyed it and glad I attended “. Most of the men (99%) responded positively to the male facilitator and this was reflected in their comments: “great to talk to another father”;” great educator, someone who has actually been there”.

Fathers in the intervention group were asked to give feedback on the educational/support materials sent to them over the first six weeks postnatal. The feedback ranged from 45%-69% (depending on the materials sent) identifying that the resources were useful /helpful. There were only 2%-8% who did not find the resources helpful.

## Discussion

Whilst childbirth does not seem to trigger long term psychological distress in most parents [62], the importance of early identification cannot be underestimated. The intervention may have contributed to the lower anxiety scores in the intervention group by providing timely, relevant information to assist the fathers with strategies for problem solving breastfeeding difficulties, sleep deprivation and “fitting” baby into their life. Stress management, lifestyle changes and the recognition of increased relationship strain are not mandatory within the antenatal program and can be completely omitted [63]. Corroborating the experience of the men in our study, a systematic review of antenatal education found that men valued access to experienced fathers and the opportunity to discuss their own concerns and learn strategies for coping with anxiety [64] The importance of information to reduce confusion and giving fathers an opportunity to explore their new roles has been identified in several studies [65,66].

The six week support package included a pamphlet on how to reduce stress and subsequent anxiety with guided imagery, muscle relaxation exercises and two herbal teabags (sent at three weeks postnatal), and only 5% of the participants indicated they did not find the relaxation exercises useful/helpful. Supporting the use of relaxation exercises to reduce anxiety, an American study with 39 psychiatric inpatients found a significant reduction in anxiety levels was obtained using relaxation exercises [67].

Process evaluation indicated fathers welcomed the information and increased input from the male facilitators that was directed at their concerns and worries. They commented that the “info was good, more than expected”. Findings from Australian and US studies support focusing on men’s needs to reduce paternal stress, improve maternal and paternal satisfaction, and enhance interpersonal skills and paternal involvement with household tasks [68,69]. Discussions around the importance of early infant contact, the different roles fathers play in the parenting arena and shared experiences by the facilitators increased the opportunity for the participants to reflect on their own fathering practice. In a similar study utilising gender specific classes with the fathers Svensson et al., (2009) found concentrating on topics such as lifestyle changes, role of the father and the importance of communication were as important as the childbearing experience [70]. Likewise, Fletcher et al., (2006) recommended that health services could better support new fathers by providing them with information on parenting from a father’s perspective, or by running father-specific sessions as part of routine antenatal care programs [11].

The study intervention’s aim was to increase the duration of breastfeeding, did not address any pre-existing conditions of depression and subsequently did not make a difference in the depression scores of the intervention fathers. Most of the fathers in both groups (95%) did not register any depression either ante or postnatal with no real changes from baseline to six weeks post test, suggesting that depression levels (5%) remained constant. In a British study 200 couples were assessed for postnatal depression at six weeks and six months post birth. Fathers were found to have 9% and 5.4% rates of PND (using the EPDS) over the two time periods [71]. Similar results from an Australian study suggest that 4% -10% of fathers suffer with postnatal depression and that many do not seek help or treatment [11]. The results from our study are consistent with the findings of a systematic review of paternal postnatal depression involving 20 international studies over 20 years to 2002, where the incidence of paternal depression ranged from 1.2% to 25.5% in community samples [72].

A past history of severe depression and high antenatal symptom scores for depression and anxiety are the strongest predictors of paternal depression in the postnatal period [37]. However, in relation to anxiety our results tend to contrast with much of the literature in that anxiety as measured by HADS reduced over time and in both intervention and control groups was approximately 2% by six weeks postnatal. While we concur with Liber et al., (2008) that it is important to reduce anxiety and depression in fathers so the parenting capacity will not be compromised and reduced by anxiety and depression [73], it is difficult to advocate for any additional resources to be allocated specifically to reduce paternal anxiety based on our results.

A limitation of this study included sampling from only public hospitals and the moderate attrition rate of 25%. Other studies have also identified moderate attrition rates with men and concluded that studies including both fathers and mothers are challenged with a generalisability problem concerning the role of the father, because there are usually increased missing data on the fathers [33,74]. Assuming a loss to follow-up of 25% in each group, 300 subjects were required in each group to be able to detect a difference at 80% power and 5% level of significance, using a Log-rank survival test. We recruited 315 fathers in the control group and 365 fathers in the intervention group, but attrition rates depleted these numbers and using only matched pairs further decreased the power of the results. The point prevalence rates at baseline and six weeks do not map anxiety and depression over time so limit the results for generalisability.

Future research needs to focus on the most effective ways to educate and inform expectant parents about the myriad of information required to facilitate a successful birth and recovery. The promising results from this small study are encouraging and further research to identify effective ways to decrease postnatal anxiety and depression are warranted. Gender specific antenatal classes could increase the engagement potential for fathers and subsequently their knowledge and support for their partner. The advances in communication technology could play a vital role in accurate, timely information for new parents: SMS, email and web based applications allow for easy access to current evidenced-based practice.

## Conclusion

Providing new fathers with timely, relevant information about breastfeeding, postnatal depression and developmental milestones for their baby may reduce perinatal anxiety and increase coping skills. Protective factors that act to reduce the risk for postnatal depression and anxiety include a parent’s possession of confidence in their abilities as a parent, good social support systems, and adequate resources for problem-solving. Improved antenatal education to meet the needs of both mothers and fathers and early awareness and intervention may limit the negative impact of perinatal anxiety and depression on parenting attitudes and behaviour. Antenatal and postnatal testing for anxiety and depression rather than being universal for all parents needs to be targeted at those parents at risk.

## Competing interests

The authors declare that they have no competing interests.

## Authors’ contributions

JT participated in the data collection and analysis and drafted the manuscript. BM has made substantial contributions to conception and design and in revising the manuscript for intellectual content and given final approval. YLH has made substantial contributions to conception and design, acquisition of data and analysis and interpretation of data; involved in manuscript revision. PH has made substantial contributions to conception and design, acquisition of data and analysis and interpretation of data; involved in manuscript revision. SB has made substantial contributions to conception and design and participated in manuscript revision. CWB has made substantial contributions to conception and design. All authors read and approved the final manuscript.

## Pre-publication history

The pre-publication history for this paper can be accessed here:

http://www.biomedcentral.com/1471-2393/12/75/prepub

## References

[B1] Buist A Austin M-P Hayes B Speelman C Bilszta J Gemmill A Brooks J Ellwood D Milgrom J Postnatal mental health of women giving birth in Australia 2002–2004: findings from the beyondblue National Postnatal Depression Program Aust N Z J Psychiatry 2008 42 1 66 73 10.1080/00048670701732749 18058446

[B2] Watanabe M Wada K Sakata Y Aratake Y Kato N Ohta H Tanaka K Maternity blues as predictor of postpartum depression: A prospective cohort study among Japanese women J Psychosom Obstet Gynaecol 2008 29 3 211 217 10.1080/01674820801990577 18608817

[B3] Leigh B Milgrom J Risk factors for antenatal depression, postnatal depression and parenting stress BMC Psychiatry 2008 8 24 10.1186/1471-244x-8-24 PMC2375874 18412979

[B4] Marcus SM Depression during pregnancy: rates, risks and consequences–Motherisk Update 2008 Can J Clin Pharmacol 2009 16 1 15 22 19164843

[B5] Marcus SM Heringhausen JE Depression in childbearing women: when depression complicates pregnancy Prim Care 2009 36 1 151 165 10.1016/j.pop.2008.10.011 19231607 PMC2680254

[B6] Grant K-A McMahon C Austin M-P Maternal anxiety during the transition to parenthood: a prospective study J Affect Disord 2008 108 1–2 101 111 18001841 10.1016/j.jad.2007.10.002

[B7] Phillips J Sharpe L Matthey S Charles M Maternally focused worry Arch Womens Ment Health 2009 12 6 409 418 10.1007/s00737-009-0091-4 19626414

[B8] Rowe H Fisher J Loh W The Edinburgh Postnatal Depression Scale detects but does not distinguish anxiety disorders from depression in mothers of infants Arch Womens Ment Health 2008 11 2 103 108 10.1007/s00737-008-0003-z 18463939

[B9] Heron J O'Connor TG Evans J Golding J Glover V The course of anxiety and depression through pregnancy and the postpartum in a community sample J Affect Disord 2004 80 1 65 73 10.1016/j.jad.2003.08.004 15094259

[B10] Matthey S Barnett B Howiec P Kavanagh DJ Diagnosing postpartum depression in mothers and fathers: whatever happened to anxiety? J Affect Disord 2003 74 2 139 147 10.1016/S0165-0327(02)00012-5 12706515

[B11] Fletcher RJ Matthey S Marley CG Addressing depression and anxiety among new fathers Medical Journal of Australia 2006 185 461 463 17137442 10.5694/j.1326-5377.2006.tb00650.x

[B12] Cowan CP Cowan PA When partners become parents: the big life change for couples 1999 Basic Books, Routledge; New York Reprinted by Lawrence Eribaum Associates

[B13] Perren S von Wyl A Bürgin D Simoni H von Klitzing K Depressive symptoms and psychosocial stress across the transition to parenthood: associations with parental psychopathology and child difficulty J Psychosom Obstet Gynaecol 2005 26 3 173 183 10.1080/01674820400028407 16295515

[B14] Buist A Morse C Durkin S Men's adjustment to fatherhood: implications for obstetric health care Journal of Obstetric, Gynaecologic, & Neonatal Nursing 2003 32 2 172 180 10.1177/0884217503252127 12685668

[B15] Condon J What about dad? Psychosocial and mental health issues for new fathers Aust Fam Physician 2006 35 9 690 692 16969437

[B16] Cabrera NJ Shannon JD Tamis-LeMonda C Fathers' Influence on Their Children's Cognitive and Emotional Development: From Toddlers to Pre-K Appl Dev Sci 2007 11 4 208 213 10.1080/10888690701762100

[B17] Fägerskiöld A A change in life as experienced by first-time fathers Scand J Caring Sci 2008 22 1 64 71 10.1111/j.1471-6712.2007.00585.x 18269424

[B18] Austin MP Tully L Parker G Examining the relationship between antenatal anxiety and postnatal depression J Affect Disord 2007 101 1–3 169 174 17196663 10.1016/j.jad.2006.11.015

[B19] O'Connor TG Heron J Glover V Antenatal anxiety predicts child behavioral/emotional problems independently of postnatal depression J Am Acad Child Adolesc Psychiatry 2002 41 12 1470 1477 10.1097/00004583-200212000-00019 12447034

[B20] Wilson S Durbin CE Effects of paternal depression on fathers' parenting behaviors: a meta-analytic review Clinical Psychological Review 2010 30 2 167 180 10.1016/j.cpr.2009.10.007 19926376

[B21] Bögels SM Bamelis L van der Bruggen CO Parental rearing as a function of parent's own, partner's, and child's anxiety status: fathers make the difference Cognition and Emotion 2008 22 3 522 538 10.1080/02699930801886706

[B22] Condon JT Boyce P Corkindale CJ The First-Time Fathers Study: a prospective study of the mental health and wellbeing of men during the transition to parenthood Aust N Z J Psychiatry 2004 38 56 64 10.1111/j.1440-1614.2004.01298.x 14731195

[B23] Zareai M O'Brien M Fallon A Creating a breastfeeding culture: A comparison of breastfeeding practises in Australia and Iran Breastfeed Rev 2008 16 1 41 43 17695073

[B24] Tohotoa J Maycock B Hauck YL Howat P Burns S Binns CW Dads make a difference: an exploratory study of paternal support for breastfeeding in Perth, Western Australia Int Breastfeed J 2009 4 15 10-1186/1746-4358-4-15 10.1186/1746-4358-4-15 PMC2788531 19943958

[B25] Nolan ML Hicks C Aims, processes and problems of antenatal education as identified by three groups of childbirth teachers Midwifery 1997 13 179 188 10.1016/S0266-6138(97)80004-6 9511685

[B26] Seligman MEP Walker EF Rosenhan DL Abnormal psychology 2001 W.W. Norton & Company, Inc, New York

[B27] Beck AT Alford B Depression Causes and Treatment 2009 University of Pennsylvania Press, Philadelphia

[B28] Hranov LG Comorbid anxiety and depression: illumination of a controversy Int J Psychiatry Clin Pract 2007 11 3 171 189 10.1080/13651500601127180 24941356

[B29] Preisig M Merikangas KR Angst J Clinical significance and comorbidity of subthreshold depression and anxiety in the community Acta Psychiatrica Scandinavia 2001 104 96 103 10.1034/j.1600-0447.2001.1040s2096.x 11473502

[B30] Gjerdingen DK Center BA First-time parents prenatal to postpartum changes in health and the relation of postpartum health to work and partner characteristics J Am Board Fam Pract 2003 16 4 304 311 10.3122/jabfm.16.4.304 12949031

[B31] Quinlivan JA Condon J Anxiety and depression in fathers in teenage pregnancy Aust N Z J Psychiatry 2005 39 10 915 920 10.1080/j.1440-1614.2005.01664.x 16168019

[B32] Skari H Skredon M Malt UF Comparative levels of psychological distress, stress symptoms, depression, and anxiety after childbirth: A prospective population-based study of mothers and fathers Bjog-International Journal ofObstetrics & Gynaecology 2002 109 10 1154 1163 10.1111/j.1471-0528.2002.00468.x 12387470

[B33] Bögels S Phares V Fathers' role in the etiology, prevention and treatment of child anxiety: A review and new model Clin Psychol Rev 2008 28 4 539 558 10.1016/j.cpr.2007.07.011 17854963

[B34] Ramchandani PG Stein A O'Connor TG Heron J Murray L Evans J Depression in men in the postnatal period and later child psychopathology: a population cohort study J Am Acad Child Adolesc Psychiatry 2008 47 4 390 398 10.1097/CHI.0b013e31816429c2 18388761 PMC2650418

[B35] Edwards B Galletly C Semmler-Booth T Dekker G Does antenatal screening for psychosocial risk factors predict postnatal depression? A follow-up study of 154 women in Adelaide, South Australia Aust N Z J Psychiatry 2008 42 1 51 55 10.1080/00048670701739629 18058444

[B36] Figueiredo B Costa R Mother’s stress, mood and emotional involvement with the infant: 3 months before and 3 months after childbirth Arch Womens Ment Health 2009 12 3 143 153 10.1007/s00737-009-0059-4 19259772

[B37] Ramchandani P G, Stein A, O'Connor TG, Heron J, Murray L, Evans J: Depression in men in the postnatal period and later child psychopathology: a population cohort study J Am Acad Child Adolesc Psychiatry 2008 47 4 390 398 10.1097/CHI.0b013e31816429c2 18388761 PMC2650418

[B38] Cox JL Holden JM Sagovsky R Detection of postnatal depression. Development of the 10-item Edinburgh Postnatal Depression Scale Br J Psychiatry 1987 150 782 786 10.1192/bjp.150.6.782 3651732

[B39] Matthey S Barnett B Ungerer J Waters B Paternal and maternal depressed mood during the transition to parenthood J Affect Disord 2000 60 2 75 85 10.1016/S0165-0327(99)00159-7 10967366

[B40] Snaith RP The Hospital Anxiety and Depression Scale Health Qual Life Outcomes 2003 1 1 29 10.1186/1477-7525-1-29 12914662 PMC183845

[B41] Johnston M Pollard B Hennessey P Construct validation of the hospital anxiety and depression scale with clinical populations J Psychosom Res 2000 48 6 579 584 10.1016/S0022-3999(00)00102-1 11033377

[B42] Martin CR Thompson DR The hospital anxiety and depression scale in patients undergoing peritoneal dialysis: internal and test-retest reliability Clin Eff Nurs 2002 6 2 78 80 10.1016/S1361-9004(02)00029-8

[B43] Bjelland I Dahl AA Haug TT Neckelmann D The validity of the Hospital Anxiety and Depression Scale; an updated review J Psychiatr Res 2002 52 69 77 10.1016/s0022-3999(01)00296-3 11832252

[B44] Robertson E Grace S Wallington T Stewart DE Antenatal risk factors for postpartum depression: a synthesis of recent literature Gen Hosp Psychiatry 2004 26 4 289 295 10.1016/j.genhosppsych.2004.02.006 15234824

[B45] Abiodun OA A validity study of the Hospital Anxiety and Depression Scale in general hospital units and a community sample in Nigeria Br J Psychiatry 1994 165 5 669 672 10.1192/bjp.165.5.669 7866683

[B46] Spinhoven PH Ormel J Sloekers PPA Kempen GIJM Speckens AEM Van Hemert AM A validation study of the Hospital Anxiety and Depression Scale (HADS) in different groups of Dutch subjects Psychol Med 1997 27 363 370 10.1017/S0033291796004382 9089829

[B47] Quinlivan JA Condon J Anxiety and depression in fathers in teenage pregnancy Aust N Z J Psychiatry 2005 39 10 915 920 10.1080/j.1440-1614.2005.01664.x 16168019

[B48] Everingham CR Heading G Connor L Couples' experiences of postnatal depression: A framing analysis of cultural identity, gender and communication Soc Sci Med 2006 62 7 1745 1756 10.1016/j.socscimed.2005.08.039 16188362

[B49] Wang S-Y Chen C-H Psychosocial health of Taiwanese postnatal husbands and wives J Psychosom Res 2006 60 3 303 307 10.1016/j.jpsychores.2005.08.012 16516664

[B50] Paulson JF Dauber S Leiferman JA Individual and combined effects of postpartum depression in mothers and fathers on parenting behaviour Pediatrics 2006 118 2 659 668 10.1542/peds.2005-2948 16882821

[B51] Ballard CG Davies R Postnatal depression in fathers Int Rev Psychiatry 1996 8 1 65 71 10.3109/09540269609037818

[B52] EscribÃ¨-AgÃ¼ir V Gonzalez-Galarzo MC Barona-Vilar C Artazcoz L Factors related to depression during pregnancy: are there gender differences? J Epidemiol Community Health 2008 62 5 410 414 10.1136/jech.2007.063016 18413453

[B53] Ramchandani PG O'Connor TG Evans J Heron J Murray LAS The effects of pre- and postnatal depression in fathers: a natural experiment comparing the effects of exposure to depression on offspring J Child Psychol Psychiatry 2008 49 10 1069 1078 10.1111/j.1469-7610.2008.02000.x 19017023 PMC2737608

[B54] O'Connor TG Annotation: The `effects' of parenting reconsidered: findings, challenges, and applications J Child Psychol Psychiatry 2002 43 5 555 572 10.1111/1469-7610.00046 12120853

[B55] Rohde P Lewinsohn PM Klein DN Seeley JR Association of parental depression with psychiatric course from adolescence to young adulthood among formerly depressed individuals J Abnorm Psychol 2005 114 3 409 420 16117578 10.1037/0021-843X.114.3.409 PMC1361262

[B56] Parker G Barnett B A test of the social support hypothesis Br J Psychiatry 1987 150 1 72 77 10.1192/bjp.150.1.72 3651719

[B57] Davey SJ Dziurawiec S O'Brien-Malone A Men's Voices: Postnatal Depression From the Perspective of Male Partners Qual Health Res 2006 16 2 206 220 10.1177/1049732305281950 16394210

[B58] Buist A Morse CA Durkin S Men's adjustment to fatherhood: implications for obstetric health care Journal of Obstetric & Gynaecological Neonatal Nursing 2003 32 2 172 180 10.1177/0884217503252127 12685668

[B59] Tohotoa J Maycock B Hauck YL Howat P Burns S Binns CW Supporting mothers to breastfeed: The development and process evaluation of a father inclusive perinatal education support program in Perth, Western Australia Health Promot Int 2011 26 3 351 361 10.1093/heapro/daq077 21156662

[B60] Nutbeam D Health promotion glossary Health Promotion International 1998 13 349 364

[B61] Lawler PA The Keys to Adult Learning: Theory and Practical Strategies 1991 Research for Better Schools, Philadelphia

[B62] Hans S Marianne S Ulrik Fredrik M Merethe D Anniken Bjornstad O Thore E Ragnhild E Comparative levels of psychological distress, stress symptoms, depression and anxiety after childbirth&;a prospective population-based study of mothers and fathers BJOG 2002 109 10 1154 1163 12387470 10.1111/j.1471-0528.2002.00468.x

[B63] Renkert S Nutbeam D Opportunities to improve maternal health literacy through antenatal education: an exploratory study Health Promot Int 2001 16 4 381 388 10.1093/heapro/16.4.381 11733456

[B64] McMillan AS Barlow J Redshaw M A Review of the Evidence about Antenatal Education 2009 Birth and Beyond, London: DH 112

[B65] Barclay L Donovan J Genovese A Men's experiences during their partner's first pregnancy: a grounded theory analysis Aust J Adv Nurs 1996 13 3 12 24 8717683

[B66] Hsin-Tzu L Kuan-Chia L Shu-Chen C Chien-Huei K Chieh-Yu L Su-Chen K A Birth Education Program for Expectant Fathers in Taiwan: Effects on Their Anxiety Birth 2009 36 4 289 296 10.1111/j.1523-536X.2009.00356.x 20002421

[B67] Weber S The Effects of Relaxation Exercises on Anxiety Levels in Psychiatric Inpatients J Holist Nurs 1996 14 3 196 205 10.1177/089801019601400303 8900613

[B68] Dellmann T "The best moment of my life": a literature review of fathers' experience of childbirth Australian Midwifery 2004 17 3 20 26 10.1016/S1448-8272(04)80014-2

[B69] Diemer GA Expectant fathers:influence of perinatal education on coping, stress, and spousal relations Res Nurs Health 1997 20 281 293 10.1002/(SICI)1098-240X(199708)20:4<281::AID-NUR2>3.0.CO;2-C 9256875

[B70] Svensson J Barclay L Cooke M Randomised-controlled trial of two antenatal education programs Midwifery 2009 25 2 114 125 10.1016/j.midw.2006.12.012 17459542

[B71] Ballard CG Davis R Cullen PC Mohan RN Dean C Prevalence of postnatal psychiatric morbidity in mothers and fathers Br J Psychiatry 1994 164 6 782 788 10.1192/bjp.164.6.782 7952984

[B72] Goodman J Paternal postpartum depression, its relationship to maternal postpartum depression, and implications for family health J Adv Nurs 2004 45 1 26 35 10.1046/j.1365-2648.2003.02857.x 14675298

[B73] Liber JM van Widenfelt BM Goedhart AW Utens EMWJ van der Leeden AJM Markus MT Parenting and Parental Anxiety and Depression as Predictors of Treatment Outcome for Childhood Anxiety Disorders: Has the Role of Fathers Been Underestimated? J Clin Child Adolesc Psychol 2008 37 747 758 10.1080/15374410802359692 18991126

[B74] Fletcher R Vimpani G Russell G Sibbritt D Psychosocial assessment of expectant fathers Archives of Womens Mental Health 2008 11 1 27 32 10.1007/s00737-008-0211-6 18246296

